# Migrainous Middle Cerebral Artery Infarction With Ipsilateral Hemiparesis

**DOI:** 10.7759/cureus.11557

**Published:** 2020-11-18

**Authors:** Taimoor A Khan, Sheharyar Zameer, Wasim Wali Muhammad, Muhammad A Zahid

**Affiliations:** 1 Internal Medicine, Headquarters Ghazaband Scouts Belleli, Quetta, PAK; 2 Medicine, Army Medical College, National University of Medical Sciences, Rawalpindi, PAK; 3 Neurology, Combined Military Hospital Multan, Multan, PAK; 4 General Medicine, Army Medical College, National University of Medical Sciences, Rawalpindi, PAK

**Keywords:** essential hypertension, homocysteine, ipsilateral hemiparesis, ischemic stroke, migrainous infarction

## Abstract

Migrainous infarction is a complication of migraine that accounts for 0.5-1.5% of all cerebral infarcts, usually seen in the posterior circulation and in young women. In this case report, we report a case of a right-sided middle cerebral artery ischemic stroke in a young male presenting with migraine, photophobia and phonophobia lasting for more than 60 minutes and followed by ipsilateral hemiparesis, which is a very unusual presentation. The provisional diagnosis of ischemic infarction of the right middle cerebral artery was made that was confirmed on radio imaging. A high index of suspicion is always required while dealing with patients with migraine especially in atypical presentations as in this case.

## Introduction

Migraine has been accepted as a complex neurological disorder as compared to the previous idea of having a vascular origin and stands as an independent risk factor for the development of infarction. It is known to have both an inherited and environmental component that emerges due to dysfunction of sensory processing of the brain [1].

Migrainous infarction is a rare complication of migraine seen as an ischemic brain lesion with onset during a ‘typical migraine with aura’ attack, usually involving the posterior circulation of the brain and occurring mostly in young women. The diagnostic criterion for migrainous infarction includes migraine with aura lasting for more than 60 minutes with a cerebral infarction of the relevant area. The exact mechanism by which the migraine progresses to the development of cerebral infarction is still not completely understood, however, it has not been found to be associated with migraine without aura [2].

Here, we present a case of migrainous infarction of the right middle cerebral artery, in a young adult male, involving the temporal, insular and parieto-occipital areas of the cerebrum and ipsilateral hemiparesis.

## Case presentation

A 31-year-old male came to the emergency unit for complaints of unilateral right-sided pulsatile headache over the last six hours preceded by phonophobia, along with drowsiness and difficulty writing. In the next 30 minutes, he developed slurring of speech and shaking of both hands. His medical history included the diagnosis of migraine with auditory and visual aura, labile hypertension, and lumbar spondylosis. The patient had a history of increased stress for the past two days. There was no history for the use of recreational drugs or smoking. Family history revealed that the father was hypertensive, and the mother had a stroke at 47 years of age.

**Figure 1:**
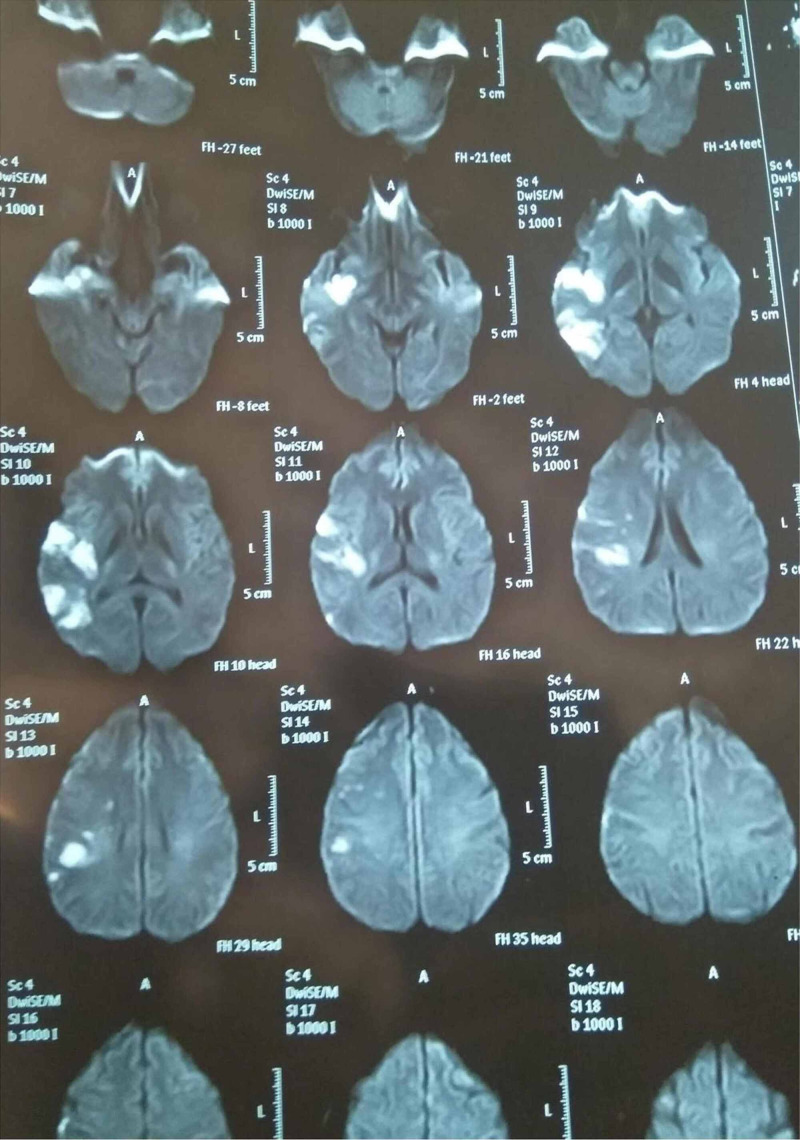
Diffusion Weighted MRI on day of admission.

On physical examination, he was nervous, obese, and in obvious distress with regular tachypnea, a blood pressure of 125/80, normal SpO2, temperature, and BSR. The neurological examination revealed a wakeful but drowsy patient. Deep tendon reflexes were preserved and symmetric while plantar reflexes were downwards going. The right upper and lower limbs showed reduced strength at 4/5 power (reduced strength against resistance) and numbness to light and gross touch. The patient was hospitalized in the medical intensive care unit (ICU) and submitted to a thorough etiological investigation. The cerebral magnetic resonance imaging (MRI) showed an area of abnormal signal intensity appearing in the right insular lobe, temporal lobe and parieto-occipital region, hypointense on T1 weighted Image and hypertensive on T2 weighted image/fluid-attenuated inversion recovery (FLAIR), as seen in Figure 1. Diffusion-weighted imaging and apparent diffusion coefficient showed restricted blood flow, suggestive of an acute infarct of the right middle cerebral artery. Thrombolytic therapy was not initiated as the window period of 4.5 hours had already lapsed. Dual antiaggregant therapy consisting of aspirin 75mg and clopidogrel 75mg was initiated. On the second day of admission the MRI Angiography suggested atherosclerotic plaque as partial opacifications within the proximal carotid arteries. The MRI Venography showed subtle voids within the transverse and sigmoid sinuses. The electrocardiogram (ECG), Troponin T and I levels, 2D echocardiogram, Exercise Tolerance Test, and Holter ECG were all normal. The electroencephalogram (EEG), carotid doppler and ultrasound (USG) kidney-ureter-bladder were also normal.

His biochemical profile including complete blood count, liver function tests, renal function tests and coagulation profile was within normal limits. Anti-HIV antibodies, hepatitis B surface antigen, and anti-hepatitis C virus antibodies were negative. Routine examination, microscopy and culture of cerebrospinal fluid (CSF) were normal. Antinuclear antibodies, anti-Sm antibodies, anti-ribonucleoprotein (RNP) antibodies, anti-Sjögren's-syndrome-related antigen A (SSA or RO) antibodies, anti-Sjogren's Syndrome B (SSB or La) antibodies, anti-Jo 1 antibodies, anti-Sci 70 antibodies, anti-neutrophil cytoplasmic autoantibodies, anti-double-stranded DNA (dsDNA) antibodies, anti-cardiolipin antibodies and rheumatoid (RA) factor were negative. Complement fraction C3 and C4 levels were normal. His 24-hour urinary vanillylmandelic acid (VMA) was normal, however serum homocysteine levels were significantly raised at 35.2 umol/L (Normal = 5-15 umol/L). Acute phase proteins, serum lactate, and muscle enzymes were normal. Serum Protein C and Antithrombin III were normal. Treponema pallidum hemagglutination test (TPHA) and Venereal Disease Research Laboratory test (VDRL) were negative.

His treatment was mainly supportive which included Aspirin 300 mg once daily, Hitop 50 mg once daily, multivitamins and a cocktail of injection Toradol 30 mg, tramadol 30 mg and dimenhydrinate 50 mg in 100 ml of normal saline twice daily for one week and then if necessary (SOS). He was also given injection magnesium sulphate 2 mg IV once daily for two weeks and then SOS. The right-sided hemiparesis gradually recovered by the sixth day of admission and the neurological exam revealed 4/5 power in left upper and lower limbs with a pronator drift in the left hand. The patient was shifted to the Medical ward and discharged on the 11th day with a weekly follow-up for up to two months and oral medicines for migraine and anti-hypertensive drugs. His homocysteine levels also gradually returned to normal during follow up. Under the clinical presentation and investigations, the patient was diagnosed as a case of migrainous infarction of the right middle cerebral artery.

## Discussion

We described a patient who had experienced a stroke for the first time after prolonged migraine with aura and the presentation of ipsilateral hemiparesis which is a rare presentation seen with middle cerebral artery ischemic infarct [3]. Our patient met all the criteria for migrainous infarction.

The corticospinal tracts (CST) are composed of two types: the lateral and anterior corticospinal tract. The lateral and anterior CST are usually involved in contralateral and unilateral motor innervation, respectively. The anterior tract usually restores the motor function when a stroke occurs in the opposite cerebral cortex however, in unusual cases, there is ipsilateral motor loss attributed to congenitally non-crossed pyramidal tracts or acquired damage to the precentral insular cortex [3].

On the other hand, the most commonly accepted hypothesis to the development of migraine is the cortical spreading depression under which a wave of rapid and nearly complete depolarization slowly propagates through the whole cortex of the brain, silencing virtually all brain activity for several minutes [4].

Migrainous infarction itself accounts for 0.5-1.5% of all ischemic strokes and does not have a completely understood origin. However, multiple possible mechanisms have been proposed including but not limited to, cortical spreading depression (CSD), biochemical alterations, hemodynamic changes, arterial vasospasm, cerebral edema, and platelet aggregation [5]. There are, however, multiple risk factors that influence the development of a cerebral infraction including smoking, heart disease, family history, etc. which were also seen in our case; homocysteinemia also exists as an independent risk factor and biomarker to the development of a cerebral stroke and needs to be investigated further [6].

The target of managing a case of migrainous infarction is to protect the brain against the acute injury while preventing more attacks in the future and if managed properly [7] the follow up of migrainous infarction is quite favorable with almost half of all patients showing complete recovery and reduction in migraine frequency after the infarction [8].

## Conclusions

The present case is of a migrainous infarction of the right middle cerebral artery presenting with ipsilateral hemiparesis, which is a rare and unusual cause of stroke in the young. Vascular changes related to cortical spreading depression, vasospasm, and hypercoagulability are postulated to cause migrainous infarct but require further investigation.
